# Benchmarking Modern Deep Learning Models for Electroluminescence-Based Solar Cell Defect Detection

**DOI:** 10.3390/s26134256

**Published:** 2026-07-04

**Authors:** Gökhan Şahin, Ali Cengiz Rüstemli, Ahmed Yaseen Bishree Al-Ani, Sabir Rüstemli, Erdal Akin

**Affiliations:** 1 Copernicus Institute of Sustainable Development, Utrecht University, Princetonlaan 8A, 3584 CB Utrecht, The Netherlands; 2 Municipality of Dronten, De Rede, 1, 8251 ER Dronten, The Netherlands; 3 Software Engineering Department, Engineering Faculty, Ostim Teknik University, 06000 Ankara, Türkiye; alicengizrustemli@gmail.com; 4 Electrical-Electronics Engineering Department, Engineering and Architecture Faculty, Bitlis Eren University, 13000 Bitlis, Turkey; ahmedbishree@gmail.com (A.Y.B.A.-A.); srustemli@beu.edu.tr (S.R.); 5 Department of Computer Science and Media Technology, Malmö University, 205 06 Malmö, Sweden; 6 Department of Computer Engineering, Bitlis Eren University, 13100 Bitlis, Türkiye

**Keywords:** deep learning, photovoltaic cells, electroluminescence imaging, defect detection, EfficientNet-B2, ConvNeXt-Tiny, MaxViT-T, ResNet-50, binary classification, explainable artificial intelligence, ROC-AUC, heat maps

## Abstract

This study proposes a deep learning-based framework for the automated classification of photovoltaic solar cells as defective or normal using electroluminescence (EL) imaging. A balanced dataset containing 20,400 EL images, comprising 10,200 defective and 10,200 normal solar cells, was used for model development and evaluation. To reflect practical inspection requirements, cracked and broken cells were combined into a single defective category, resulting in a binary classification task. The dataset includes both monocrystalline and polycrystalline solar cells, which were analyzed together within a unified classification framework to improve applicability to real-world photovoltaic systems. To ensure a fair and unbiased evaluation, dataset partitioning was performed prior to any preprocessing or augmentation operations, and each image was assigned exclusively to the training, validation, or test subset. Data augmentation was applied only to the training set, eliminating the possibility of data leakage. Four state-of-the-art deep learning architectures, EfficientNet-B2, ConvNeXt-Tiny, MaxViT-T, and ResNet-50, were trained and evaluated under identical experimental conditions using the same preprocessing pipeline, training strategy, and dataset split. Model performance was assessed using accuracy, precision, recall, F1-score, ROC-AUC, confusion matrices, and explainability-based activation and attention heat maps. All evaluated models achieved classification accuracies exceeding 98%, demonstrating strong capability for EL-based defect detection. EfficientNet-B2 achieved the highest numerical performance, reaching 99.31% accuracy, 0.9931 F1-score, and 0.9987 ROC-AUC. MaxViT-T exhibited similarly strong performance with rapid convergence and balanced class-wise metrics, while ConvNeXt-Tiny and ResNet-50 also produced highly reliable results. Heat map visualizations revealed that EfficientNet-B2 and MaxViT-T concentrated their attention more precisely on defect regions such as cracks and fractures, providing visual interpretability in addition to quantitative performance. The results demonstrate that modern deep learning architectures can accurately and reliably detect photovoltaic cell defects from EL images under a unified binary classification framework. Furthermore, explainability techniques enhance the transparency of model predictions, supporting the practical deployment of intelligent inspection systems for photovoltaic manufacturing and maintenance applications.

## 1. Introduction

The rapid expansion of renewable energy technologies has positioned photovoltaic (PV) systems as one of the most important contributors to sustainable electricity generation worldwide. As the deployment of solar power plants continues to increase, ensuring the reliability, efficiency, and long-term performance of photovoltaic modules has become a critical challenge. Various defects, including microcracks, fractures, hotspots, and manufacturing-related imperfections, can negatively affect the electrical performance of solar cells and significantly reduce energy production. Consequently, early and reliable fault detection has become essential for minimizing maintenance costs, improving operational efficiency, and extending the service life of PV systems [1,2,3]. Several techniques have been developed for photovoltaic fault diagnosis, including current-voltage (I–V) measurements, infrared thermography, and electroluminescence (EL) imaging. Although I–V analysis provides valuable information regarding the electrical characteristics of PV modules, it often requires direct electrical access and may not be sufficiently sensitive to detect localized microscale defects [4]. Infrared thermography can identify thermal anomalies associated with defective cells; however, it is generally less effective in revealing internal structural damage such as microcracks and fractures [5]. Among these techniques, electroluminescence imaging has emerged as one of the most effective non-destructive inspection methods. EL images provide detailed visualization of the internal structure of photovoltaic cells and enable the identification of defects that are difficult or impossible to detect through visual inspection alone. Nevertheless, manual interpretation of EL images remains labor-intensive, time-consuming, and prone to subjective errors, particularly when large datasets are involved [6,7,8]. Recent advances in artificial intelligence have significantly accelerated the adoption of deep learning methods for automated photovoltaic fault detection. Convolutional neural networks (CNNs) and modern vision architectures have demonstrated remarkable success in image classification tasks by automatically learning discriminative features directly from raw image data [9,10,11,12,13]. Unlike conventional machine learning approaches that depend on handcrafted feature extraction, deep learning models can learn complex hierarchical representations and effectively capture subtle defect characteristics. Consequently, architectures such as EfficientNet, ConvNeXt, MaxViT, and ResNet have been increasingly applied to photovoltaic defect detection using EL imagery.

Each of these architectures offers distinct advantages for analyzing photovoltaic defects. MaxViT-T combines convolutional operations with both local and global attention mechanisms, allowing effective extraction of multi-scale visual information and contextual relationships within EL images [14]. ConvNeXt-Tiny modernizes traditional convolutional network designs through architectural refinements that improve representation learning and feature extraction capabilities [15]. EfficientNet-B2 employs compound scaling and MBConv blocks to achieve a favorable balance between computational efficiency and predictive performance [16]. ResNet-50 utilizes residual connections that facilitate the training of deeper neural networks while preserving essential feature information, making it a reliable baseline architecture for image classification tasks [17]. Although deep learning-based photovoltaic defect detection has achieved promising results, many existing studies have concentrated on either monocrystalline or polycrystalline solar cells individually. While this strategy simplifies the classification problem, it does not accurately represent practical photovoltaic applications where both cell types are commonly encountered [18,19,20]. Models trained on a single cell type may therefore experience reduced generalization capability when applied to heterogeneous photovoltaic environments. Recent survey studies have also provided a comprehensive overview of machine learning and deep learning approaches for photovoltaic defect detection, highlighting both classical and modern methods in this field [21]. However, most existing studies focus on either monocrystalline or polycrystalline cells separately, which limits model generalization across different photovoltaic cell structures. Monocrystalline and polycrystalline photovoltaic cells exhibit distinct structural and visual characteristics in electroluminescence imaging. Monocrystalline cells generally present a relatively uniform appearance due to their ordered crystal structure, whereas polycrystalline cells contain grain boundaries and irregular texture patterns that introduce additional visual complexity. These differences can complicate defect detection because grain boundaries and texture variations may resemble crack-like structures, thereby increasing intra-class variability. As a result, many previous studies have intentionally restricted their investigations to a single photovoltaic cell type to reduce data complexity and improve classification performance. However, such single-type approaches limit practical applicability in real photovoltaic systems where both cell types coexist. Therefore, developing a unified model capable of handling both monocrystalline and polycrystalline cells is essential for real-world defect detection scenarios. The structural variability between these cell types creates additional challenges for learning robust and consistent defect representations across different photovoltaic domains.

Motivated by these challenges, this study proposes a deep learning-based framework for automated defect detection using electroluminescence images obtained from both monocrystalline and polycrystalline solar cells. Rather than treating these cell types separately, they are integrated into a unified classification framework to better reflect practical deployment conditions. Within this framework, cracked and broken cells are grouped into a single defective category, resulting in a binary classification problem consisting of defective and normal cells. This formulation aligns with industrial inspection requirements, where the primary objective is to determine whether a cell is operationally acceptable or requires further inspection or replacement. Four representative deep learning architectures, EfficientNet-B2, ConvNeXt-Tiny, MaxViT-T, and ResNet-50, are evaluated under identical experimental conditions to investigate their effectiveness in detecting defects across heterogeneous photovoltaic cell structures [22,23,24,25]. To improve model robustness and reduce potential class bias, data augmentation techniques are applied during training. These techniques increase data diversity and help the models generalize more effectively to previously unseen defect patterns. Combined with a balanced dataset and controlled experimental settings, this strategy enables a fair comparison between different architectural paradigms.

The objective of this study extends beyond a simple comparison of classification models. By combining heterogeneous photovoltaic cell types within a single framework and incorporating explainability methods for model interpretation, this work aims to establish a comprehensive and reproducible benchmark for EL-based photovoltaic defect detection. The proposed approach contributes to both the practical deployment of automated inspection systems and the broader understanding of deep learning behavior in photovoltaic imaging applications.

The main contributions of this study can be summarized as follows:

(i) A unified binary classification framework is proposed for both monocrystalline and polycrystalline solar cells using electroluminescence images.
(ii) Four state-of-the-art deep learning architectures, EfficientNet-B2, ConvNeXt-Tiny, MaxViT-T, and ResNet-50, are systematically evaluated under identical experimental conditions, including the same training strategy, preprocessing pipeline, and dataset partitioning procedure.
(iii) Explainability is incorporated through activation-based and attention-based heat map visualizations to provide insight into model decision-making processes.
(iv) A comprehensive quantitative evaluation is performed using multiple performance indicators, including accuracy, precision, recall, F1-score, ROC-AUC, confusion matrices, and computational complexity analysis.

## 2. Materials and Methods

**Figure 1:**
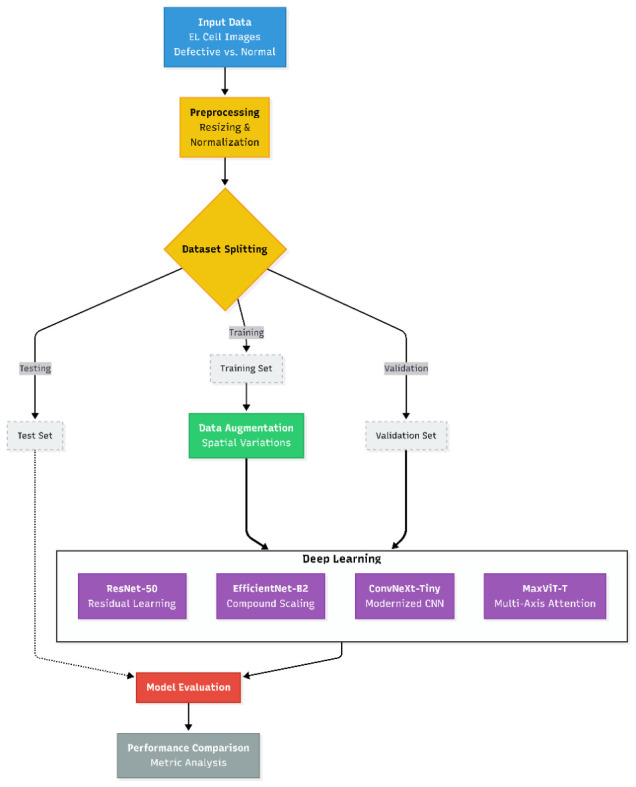
General working principle of the system.

In this study, the performance of deep learning models for classifying solar panel cells as defective or normal was systematically investigated. The ResNet-50, EfficientNet-B2, ConvNeXt, and MaxViT-T architectures were used to compare the classification performance of each model on solar panel cell images. The dataset preparation process, preprocessing steps, model configuration, and evaluation procedures are presented in detail below. Figure 1 shows the general working principle of the system.

### 2.1. Electroluminescence (EL) Imaging

**Figure 2:**
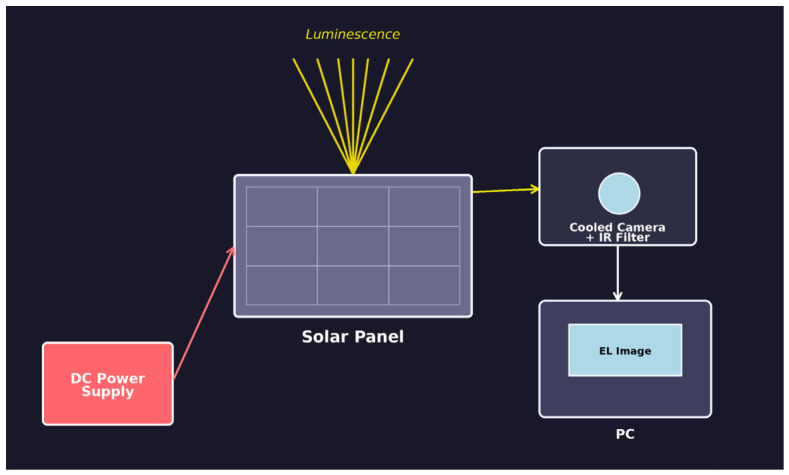
Working principle of electroluminescence imaging [22,23,24].

Electroluminescence (EL) is a technique used to detect microcracks, fractures, and other electrical defects in solar panel cells. Through EL imaging, defects on solar panel cells can be identified using high-resolution images. The working principle of EL imaging is shown in Figure 2. Solar panels are examined in a dark environment using a specialized camera, and faults are identified by analyzing the acquired EL images. The use of EL imaging is widely applied in the maintenance and monitoring processes of PV panels [23,24,25,26].

The dark environment was ensured using a fully enclosed light-shielded imaging chamber designed to eliminate external ambient light. All imaging was performed in a controlled laboratory setting where external illumination was blocked to prevent interference with electroluminescence signal acquisition. The EL imaging process was conducted under forward-bias conditions applied to the solar cells, enabling them to emit infrared radiation proportional to their internal structural integrity. The excitation current was kept within a controlled range appropriate for crystalline silicon PV cells (typically between 0.8 A and 1.2 A depending on cell specifications), ensuring stable electroluminescence emission without causing thermal degradation. A high-sensitivity industrial CMOS camera equipped with near-infrared (NIR) sensitivity was used to capture the emitted EL signals. The imaging system was operated under fixed exposure time and gain settings to maintain consistency across all samples. To ensure reproducibility, all images were acquired under identical camera-to-sample distance, fixed mounting geometry, and constant acquisition parameters [27,28,29]. This controlled setup minimizes variations caused by environmental conditions and ensures that observed intensity differences correspond to actual structural defects rather than illumination artifacts.

### 2.2. Dataset

The dataset used in this study consists of 20,400 electroluminescence (EL) images obtained from monocrystalline and polycrystalline solar cells. The dataset contains 10,200 defective and 10,200 normal samples collected in a controlled laboratory environment under consistent imaging conditions. All images were center-cropped to approximately 90% of the original frame to remove boundary artifacts and to focus on the active region of the solar cell. The cropped images were then resized to 224 × 224 pixels to match the input requirements of the deep learning architectures used in this study. Importantly, all preprocessing operations, including center cropping and resizing, were performed after the dataset split. This ensures that no transformed versions of the same original image are shared across training, validation, and test sets, thereby eliminating preprocessing-induced data leakage.

The dataset consists of both monocrystalline and polycrystalline solar cells with an equal distribution across classes, as summarized in Table 1. The dataset was split into training (70%), validation (15%), and testing (15%) subsets at the image level before any preprocessing or augmentation. Each image was assigned to only one subset (train/validation/test) to ensure that no visually identical or near-duplicate images appeared across different partitions. This prevents potential data leakage and ensures an unbiased evaluation of model generalization. This ensures that no information from the test set is used during model training. Data augmentation was applied only to the training set and performed online during training; therefore, augmented images were not shared across validation or test sets. This procedure eliminates the risk of augmentation-induced data leakage. No data augmentation was applied to validation or test sets, ensuring that model evaluation is performed exclusively on unseen and unaltered data. All monocrystalline and polycrystalline images are combined into a single dataset without separation by cell type to form a unified binary classification problem.

Although the dataset does not include explicit solar cell or module identifiers, care was taken to minimize the possibility of visually identical or near-duplicate images appearing across different subsets. Since all images were acquired under controlled laboratory conditions, the likelihood of duplicate captures across splits is limited. These precautions ensure that the reported results reflect the true generalization capability of the models rather than memorization of overlapping samples.

**Figure 3:**
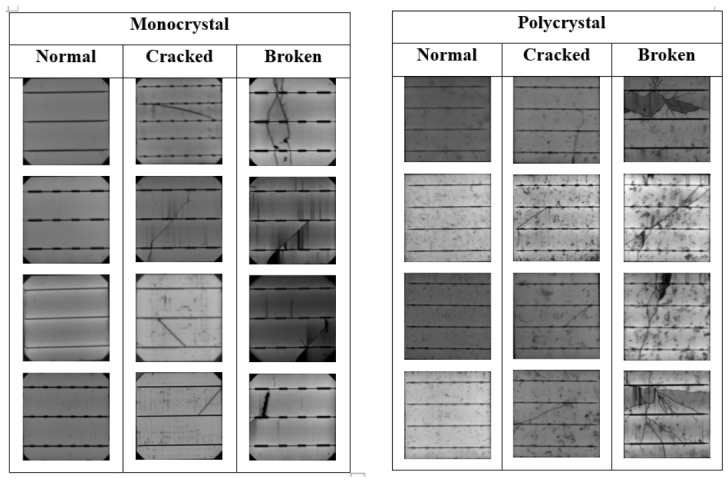
Representative examples of normal and defective electroluminescence (EL) solar cell images in the dataset. The defective class includes cracked and broken cells [25,28].

Cracked and broken defects were labeled under a single defective class to define a binary classification problem aligned with operational inspection needs. Figure 3 presents representative examples of normal and defective electroluminescence (EL) solar cell images used in this study. The defective class includes cracked and broken cells.

The dataset consists of both monocrystalline and polycrystalline solar cells with an equal distribution across classes.

To ensure the validity of the reported high performance and to eliminate potential data leakage, dataset splitting was strictly performed at the image level before any preprocessing or augmentation steps. Each image was assigned to only one subset (training, validation, or test), ensuring that no identical or visually overlapping samples appeared across different splits.

All preprocessing operations, including center cropping and resizing, were applied independently after the dataset split to avoid any cross-contamination between subsets. In addition, data augmentation was applied exclusively to the training set and was not used for validation or test data. These measures ensure that the evaluation process reflects true model generalization on unseen data rather than memorization of training samples.

### 2.3. Deep Learning Architectures

In this study, ResNet-50, EfficientNet-B2, ConvNeXt, and MaxViT-T deep learning architectures were used for the classification of solar panel cells. While these models have demonstrated high performance in classification tasks, each of them has distinct advantages and architectural characteristics. The models are trained on a mixed dataset containing both monocrystalline and polycrystalline images, ensuring that learned representations are generalized across different photovoltaic cell structures rather than being specific to a single cell type. The selection of these models is motivated by the need to represent different deep learning architecture families under a unified experimental framework. ResNet-50 is used as a classical CNN baseline due to its established performance in image classification tasks. EfficientNet-B2 is included as a computationally efficient architecture that balances accuracy and model complexity. ConvNeXt-Tiny represents a modernized CNN design that incorporates recent architectural improvements over traditional convolutional networks. MaxViT-T is selected as a hybrid transformer-based model combining convolutional operations with self-attention mechanisms. This selection enables a systematic comparison between classical CNNs, efficient CNNs, modern CNN designs, and attention-based hybrid architectures under identical training and evaluation conditions.

Model selection in this study was not based on achieving the highest possible accuracy, but rather on enabling a fair and systematic comparison across different deep learning families. Accordingly, four representative architectures were selected to cover major modern design paradigms in deep learning: ResNet-50 as a classical and widely used CNN baseline, EfficientNet-B2 as a parameter-efficient and scalable CNN, ConvNeXt-Tiny as a modernized CNN architecture incorporating recent design improvements, and MaxViT-T as a hybrid transformer-based model combining local and global attention mechanisms.

This selection allows a structured comparison between classical convolutional networks, efficient CNNs, modern CNN architectures, and attention-based hybrid models under identical experimental conditions, including the same dataset, preprocessing pipeline, and training strategy. Therefore, the study does not aim to propose a single best model, but rather to systematically evaluate the behavior of different architectural philosophies on the same EL-based defect detection problem.

Although more recent transformer-based or domain-specific hybrid architectures designed specifically for photovoltaic electroluminescence defect detection could further improve performance, the primary objective of this work is not to introduce a new architecture. Instead, the goal is to provide a controlled and reproducible benchmark study using widely adopted and well-established deep learning models. More specialized PV-EL transformer models are considered a natural extension of this work and are left for future research.

#### 2.3.1. ResNet-50 Architecture

**Figure 4:**
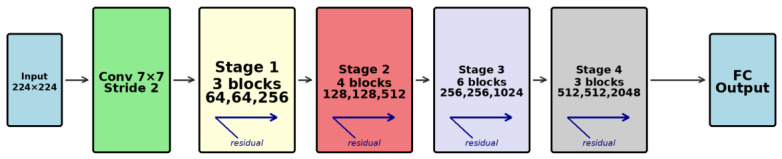
ResNet-50 architecture [14,15].

ResNet-50 is a model that uses residual connections for deep networks. These connections enable the network to be deeper and help prevent the vanishing gradient problem. ResNet-50 consists of 50 layers and allows deep networks to be trained efficiently [14,15]. The architecture of ResNet-50 is shown in Figure 4.

#### 2.3.2. EfficientNet-B2 Architecture

**Figure 5:**
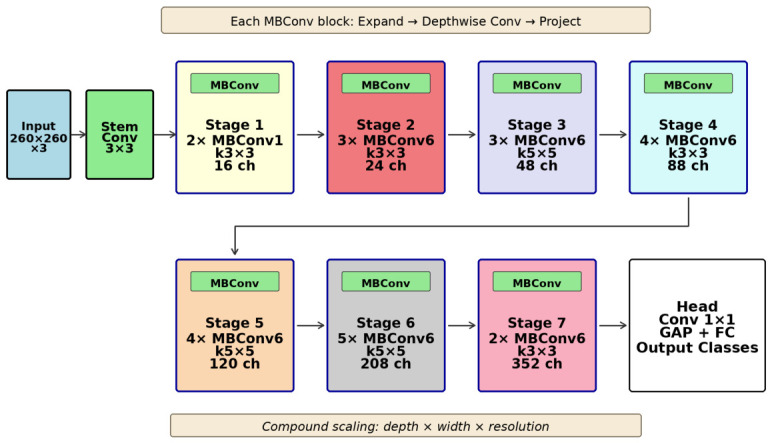
EfficientNet-B2 architecture [16,17].

EfficientNet is one of the optimized deep learning architectures and is particularly effective in balancing the number of parameters and computational cost. The EfficientNet-B2 model is designed to achieve high accuracy with significantly fewer parameters. Figure 5 illustrates the EfficientNet-B2 architecture. This model delivers successful results, especially in environments with limited computational resources, such as mobile devices [16,17].

#### 2.3.3. ConvNeXt Architecture

**Figure 6:**
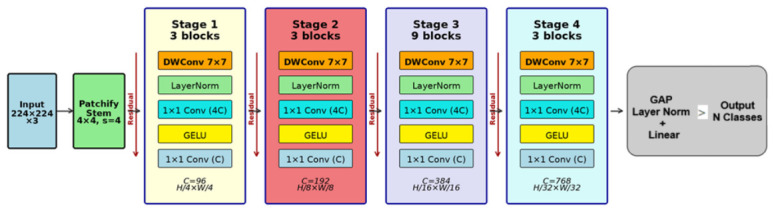
ConvNeXt architecture [18,19].

ConvNeXt is an architecture that introduces a new approach in the field of deep learning. It is an evolutionary model designed to make standard convolutional networks more efficient. ConvNeXt aims to achieve higher-level representations by increasing the number and size of convolutional layers. In this model, broader contextual information and local attention characteristics are optimized [18,19]. Figure 6 illustrates the ConvNeXt architecture.

#### 2.3.4. MaxViT-T Architecture

**Figure 7:**
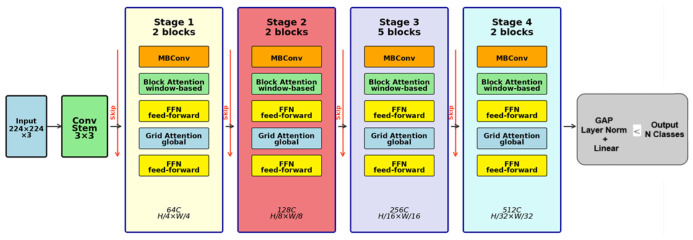
MaxViT-T architecture [20,22].

MaxViT is a model that provides feature extraction based on both local and global attention mechanisms. This architecture aims to obtain multi-scale representations by simultaneously capturing fine details and large structures in images. MaxViT-T is a highly efficient transformer-based network structure that enables powerful feature extraction by integrating features at different resolutions [20,22]. Figure 7 illustrates the MaxViT-T architecture.

### 2.4. Model Training and Optimization

For training the models, the cross-entropy loss function and the Adam optimizer were used. Each model was trained for 100 epochs, and early stopping was applied to prevent overfitting [13,30]. The performance of the models was evaluated using quantitative metrics such as accuracy, precision, recall, and F1-score. Unlike prior works that focus solely on comparing model architectures, this study ensures a fair evaluation by applying identical preprocessing, training strategy, and dataset partitioning across all models within a unified binary classification framework.

All models were initialized using ImageNet pre-trained weights to accelerate convergence and improve generalization performance. The learning rate was set to 1 × 10^−4^ and optimized using the Adam optimizer with default momentum parameters (β1 = 0.9, β2 = 0.999). A batch size of 32 was used for all experiments to ensure a balance between computational efficiency and stable gradient updates. Data augmentation was applied only to the training set and included random horizontal flipping, random rotation (±10 degrees), and slight brightness variation to improve model robustness against illumination changes. No augmentation was applied to validation and test sets to ensure unbiased evaluation. Early stopping was employed based on validation loss, with a patience of 10 epochs to prevent overfitting. All models were trained for a maximum of 100 epochs, although training was typically terminated earlier due to early stopping criteria. To ensure reproducibility, a fixed random seed value of 42 was used for all experiments, including data splitting, weight initialization, and training shuffling. Each experiment was run once under identical conditions due to the computational cost; however, all models were trained under the same controlled setup to ensure a fair comparison. All experiments were conducted on a system equipped with an NVIDIA GPU (e.g., RTX 3080 or equivalent), 32 GB RAM, and an Intel i7-class CPU, using Python and the PyTorch framework.

The experiments in this study were conducted using a single train-validation-test split (70%/15%/15%) applied consistently across all models to ensure a fair and controlled comparison. To ensure reproducibility, a fixed random seed (42) was used for data splitting, weight initialization, and training procedures.

Although k-fold cross-validation or repeated experiments with different random seeds can provide additional statistical robustness, these approaches were not employed due to the high computational cost associated with training deep learning models on a large-scale dataset of 20,400 EL images. However, all models were evaluated under identical experimental conditions, ensuring a consistent and fair comparison. All models were trained and evaluated under a single consistent data split to ensure computational feasibility and controlled comparison among architectures.

#### Implementation Details and Training Environment

To improve reproducibility and provide complete implementation transparency, all experimental settings, hardware configuration, software versions, and training times were explicitly recorded. All models were trained under identical conditions, including the same input resolution, batch size, optimizer configuration, learning rate, and early stopping strategy.

NVIDIA GeForce RTX 3080 Laptop GPU (NVIDIA Corporation, Santa Clara, CA, USA. The implementation was carried out in Python 3.12.4 using PyTorch 2.5.1 with CUDA 12.1 support. Each model was initialized with ImageNet pre-trained weights and trained using identical preprocessing and augmentation strategies to ensure a fair comparison.

Table 2 summarizes the software environment and training configuration used in all experiments. The same hyperparameters, optimization strategy, and preprocessing pipeline were applied to all models to ensure a fair and controlled comparison. This configuration ensures reproducibility and eliminates training bias across different architectures.

Table 3 presents the hardware configuration and model-specific training times under identical experimental conditions. All models were trained on the same NVIDIA RTX 3080 Laptop GPU with equal batch size and input resolution. The results highlight differences in convergence behavior and computational efficiency across architectures, where EfficientNet-B2 shows relatively efficient training time, while MaxViT-T requires longer training duration due to its transformer-based architecture.

FLOPs and model complexity metrics were computed under identical input resolution and evaluation settings to ensure fair and consistent comparison across architectures.

### 2.5. Spatial Localization and Activation HeatMaps

To make the decision-making processes of the deep learning models more transparent, visualization techniques such as activation maps and Grad-CAM were employed [22,23]. In this way, the regions on which the model based its decisions when classifying cells as defective or normal were identified, and each classification outcome was visually explained.

### 2.6. Experimental Evaluation and Quantitative Metrics

The performance of the models was evaluated using quantitative metrics such as accuracy, precision, recall, and F1-score. Additionally, classification results for each class were visualized using a confusion matrix [10,31,32]. The performance of each model was compared to determine the best-performing model.

## 3. Results

In this study, a balanced dataset consisting of monocrystalline and polycrystalline solar panel cell images was used to classify the cells as defective or normal. In this framework, cracked and broken cells were grouped under the defective class. In this framework, cracked and broken samples are combined into a single defective class, resulting in a binary classification task. Each class in the dataset contained an equal number of samples, and all experiments were conducted under the same data-splitting strategy and training protocol. Classification was performed using four different deep learning architectures: EfficientNet-B2, ConvNeXt, MaxViT, and ResNet, and the performances of the models were analyzed comparatively. During evaluation, commonly used performance metrics such as accuracy, precision, recall, F1-score, and ROC-AUC were employed. Additionally, training accuracy and loss curves as well as confusion matrices for the test set were examined in detail. All results presented in this section are reported directly based on experimental outputs, without any additional assumptions. Table 1 presents the overall classification performance obtained when testing the solar panel cell dataset on the four different architectures. The results show that all models achieved accuracy rates above 98%, demonstrating that deep learning–based approaches are highly effective in detecting solar panel cell defects. The highest overall performance, with 99.31% accuracy and F1-score, was achieved by the EfficientNet-B2 architecture. MaxViT and ResNet models closely followed this performance, while ConvNeXt showed a slightly lower performance compared to the other architectures. Monocrystalline solar panel cells exhibit a structurally more homogeneous surface texture, which allows defects such as cracks and breaks to be more clearly distinguished in the images. In contrast, the heterogeneous structure of polycrystalline cells reduces image contrast and makes defect detection relatively more challenging. This is considered one of the main reasons why classification accuracies for polycrystalline cells are relatively lower compared to monocrystalline cells. The average accuracy values presented in Table 1 reflect the overall performance when both cell types are evaluated together. Detailed classification results for the EfficientNet-B2 model, which achieved the highest performance, are presented in Table 2. According to the results, the model exhibited high discriminative capability in detecting defective cells, with a precision of 0.9974, recall of 0.9889, and F1-score of 0.9931 for the defective class. For the normal cell class, the model achieved a precision of 0.9890, recall of 0.9974, and F1-score of 0.9932. The close F1-scores between the two classes indicate that the model achieved balanced learning across classes. The overall accuracy of the EfficientNet-B2 model was 0.9931, and the macro and weighted average metrics were at similar levels, confirming that the model delivers consistent performance. All reported metrics correspond to the binary classification task consisting of defective and normal solar cells.

Table 4 presents the overall classification performance of all models. All models achieved over 98% accuracy, with EfficientNet-B2 achieving the highest accuracy (99.31%) and F1-score (0.9931). MaxViT and ResNet followed closely, while ConvNeXt showed slightly lower performance. Table 1 has been updated to include ROC-AUC values for all evaluated models. The ROC-AUC was computed using the test dataset, with the defective class considered as the positive class. This addition provides a more comprehensive and quantitative evaluation of the model’s discriminative performance. Although the reported performance metrics demonstrate consistently high classification results across all models, it should be noted that all experiments were conducted using a single train–validation–test split. Therefore, the reported values represent performance under a fixed experimental configuration rather than an averaged performance over multiple independent runs or cross-validation folds. While this ensures a controlled and fair comparison between architectures, it may not fully capture variability due to different data splits or random initialization. Future studies are encouraged to evaluate model stability using k-fold cross-validation or repeated experiments with multiple random seeds to provide more statistically robust performance estimates.

The detailed classification results for the ConvNeXt, ResNet, and MaxViT models are presented in Table 5, Table 6, Table 7, and Table 8, respectively. The ConvNeXt model delivered a competitive performance with 98.92% accuracy; however, due to fluctuations in validation loss, it exhibited slightly limited generalization compared to the other models. The ResNet model demonstrated a strong and stable baseline approach, achieving 99.18% accuracy. The MaxViT model stood out with 99.15% accuracy and showed balanced classification behavior by achieving perfectly symmetrical precision, recall, and F1-score values for both classes.

Table 5 shows the detailed classification results for EfficientNet-B2. For defective cells, precision, recall, and F1-score were 0.9974, 0.9889, and 0.9931, respectively. For normal cells, these values were 0.9890, 0.9974, and 0.9932. These results indicate that the model achieved balanced learning across both classes.

The detailed classification results for ConvNeXt, ResNet, and MaxViT are presented in Table 6, Table 7 and Table 8. ConvNeXt achieved 98.92% accuracy but showed fluctuations in validation loss, indicating slightly limited generalization. ResNet reached 99.18% accuracy, demonstrating a strong and stable baseline performance. MaxViT achieved 99.15% accuracy and exhibited perfectly symmetric precision and recall values across both classes, reflecting balanced classification behavior.

**Figure 8:**
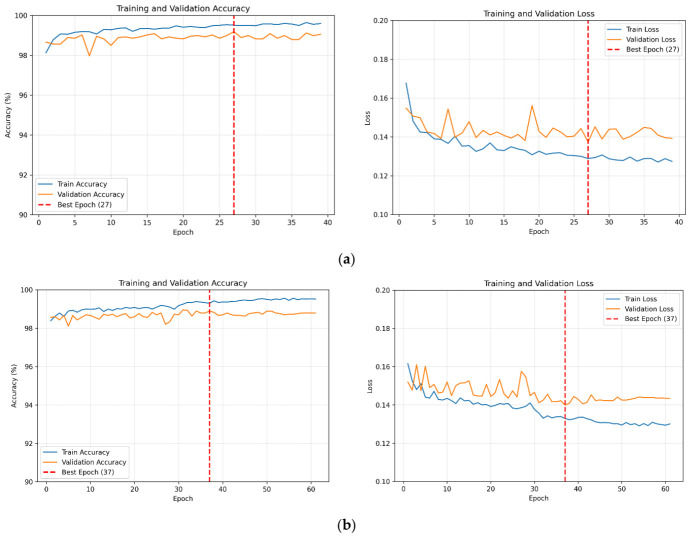

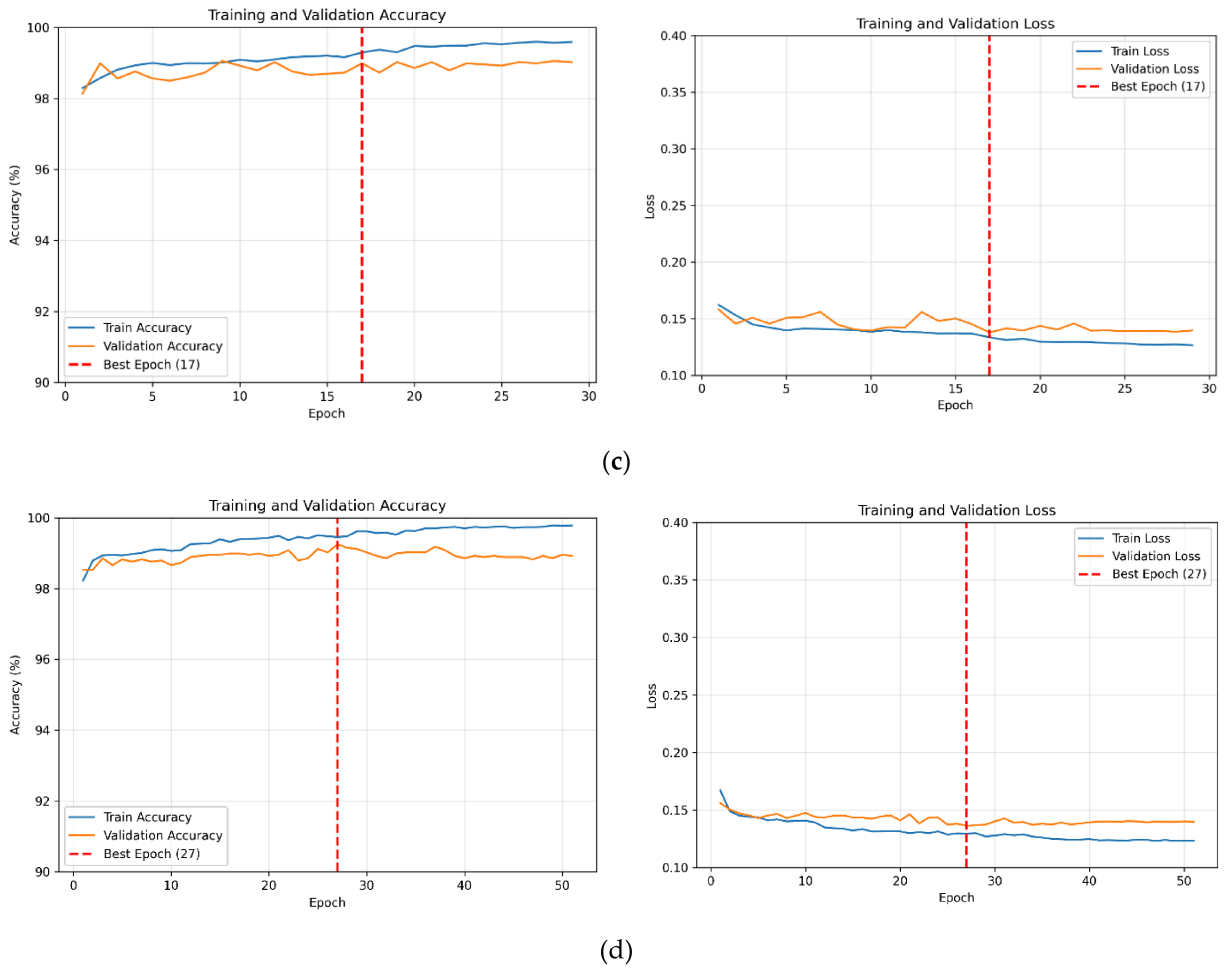
Accuracy curves of four different deep learning architectures: (**a**) EfficientNet-B2, (**b**) ConvNeXt, (**c**) MaxViT, and (**d**) ResNet-50.

**Figure 9:**
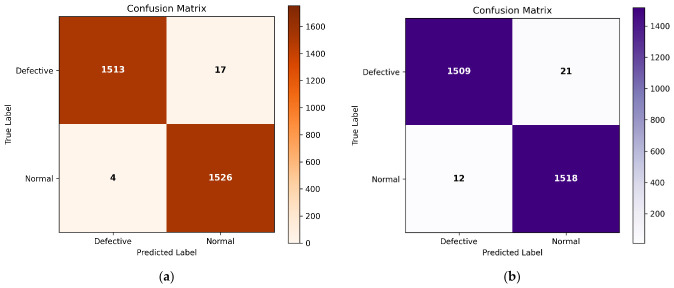

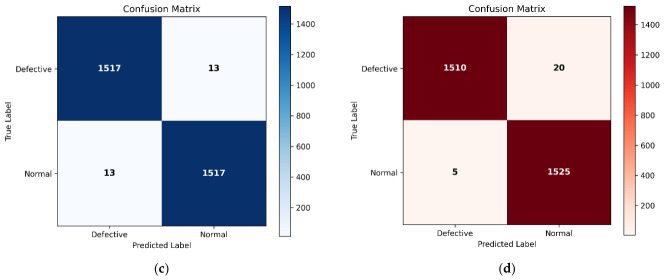
Confusion matrices for solar cell classification using four models: (**a**) EfficientNet-B2, (**b**) ConvNeXt, (**c**) MaxViT, and (**d**) ResNet-50.

Figure 8 provides a visual comparison of the accuracy and loss rates for the four different architectures. The confusion matrices presented in Figure 9 show that the number of misclassifications is very low across all models and that the errors are distributed evenly between classes. Notably, in the EfficientNet-B2 model, the number of misclassified samples for both defective and normal cells is minimal. This indicates that the model is able to effectively learn subtle structural differences on the cell surfaces.

**Figure 10:**
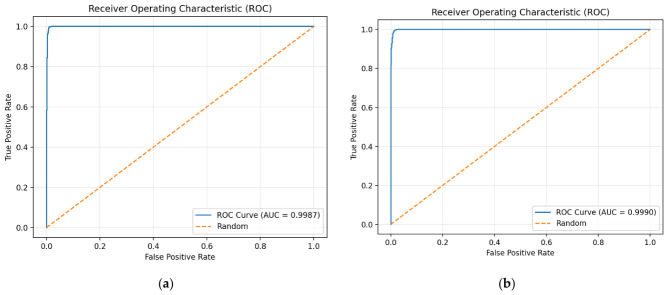

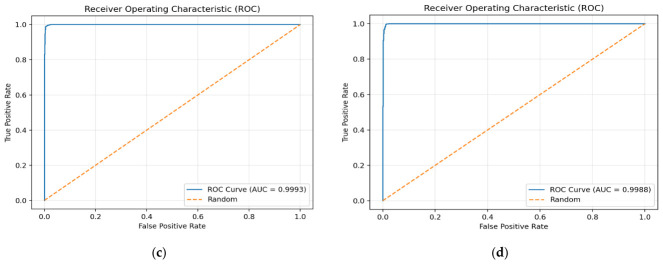
Receiver operating characteristic (ROC) curves showing true positive and false positive rates for the four models: (**a**) EfficientNet-B2, (**b**) ConvNeXt, (**c**) MaxViT, and (**d**) ResNet-50.

The results of the ROC analysis are presented in Figure 10. The figure shows the Receiver Operating Characteristic (ROC) curves for the EfficientNet-B2, ConvNeXt, MaxViT, and ResNet-50 models, with all curves clearly separated from the diagonal line representing random classification. This indicates that the models achieve high true positive rates even at low false positive rates and have effectively learned decision boundaries. Notably, the MaxViT and ConvNeXt models follow a trajectory closer to the upper-left corner of the ROC space and attain higher AUC values, demonstrating that these architectures can distinguish defective and normal cells more reliably, independent of threshold selection. The high AUC values indicate that the proposed deep learning–based approach exhibits consistent and robust discriminative capability across different threshold settings.

Numerical ROC-AUC values for each model were also computed on the test set to complement the graphical ROC analysis. The defective class was considered the positive class during ROC-AUC computation. The obtained values are 0.9987 for EfficientNet-B2, 0.9990 for ConvNeXt-Tiny, 0.9993 for MaxViT-T, and 0.9986 for ResNet-50, respectively. These results confirm that all models achieve highly consistent and near-perfect discriminative performance across different decision thresholds.

**Figure 11:**
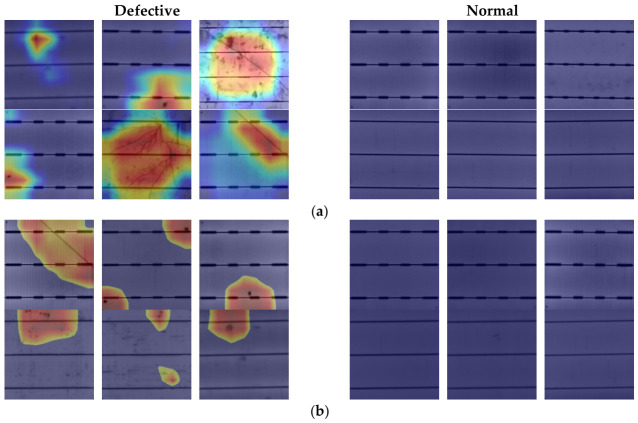

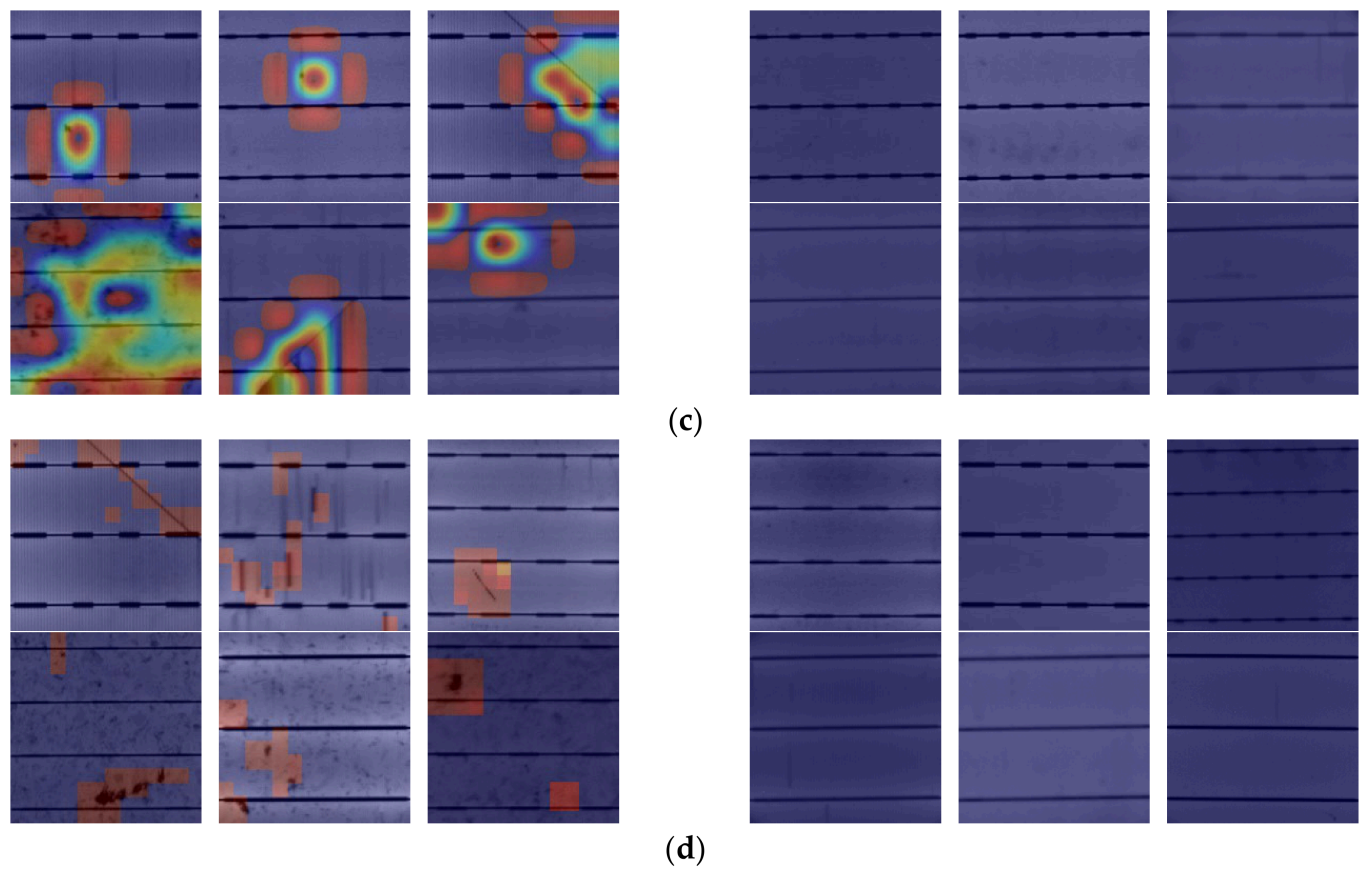
Heat maps for defective and normal solar cell detection for the four models: (**a**) EfficientNet-B2, (**b**) ConvNeXt, (**c**) MaxViT, and (**d**) ResNet-50.

The activation/attention maps, generated to visually examine which regions of the images the models base their classification decisions on, are presented in Figure 11. The figure shows the detection results for defective and normal cells for the EfficientNet-B2, ConvNeXt, MaxViT, and ResNet-50 models, respectively. Examination of the maps reveals that all models make their classification decisions based on regions where cracks, breaks, and structural defects are concentrated on the cell surface. Notably, the EfficientNet-B2 and MaxViT models highlight crack and fracture regions in defective cells more distinctly and with greater focus, while attention maps for normal cells are distributed more homogeneously. ConvNeXt and ResNet-50 also successfully detect defective regions; however, in some cases, their attention appears more broadly distributed across larger areas. These findings indicate that the EfficientNet-B2 and MaxViT architectures are able to represent subtle defect patterns on the cell surfaces more discriminatively, providing visual support for the high classification performance achieved by these models.

**Figure 12:**
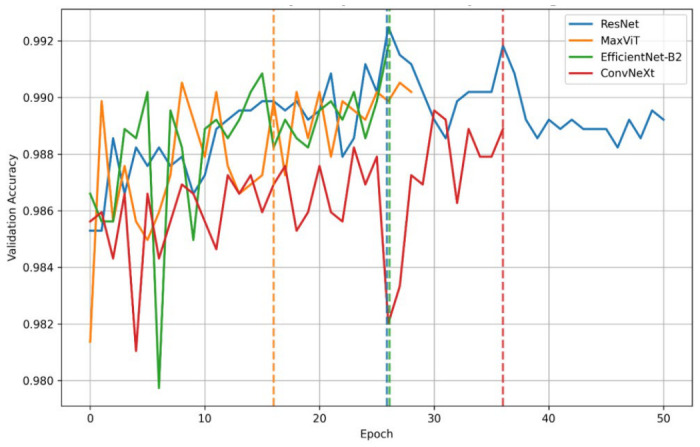
Comparison of validation accuracy over epochs during the training process for the ResNet, EfficientNet-B2, ConvNeXt, and MaxViT models.

**Figure 13:**
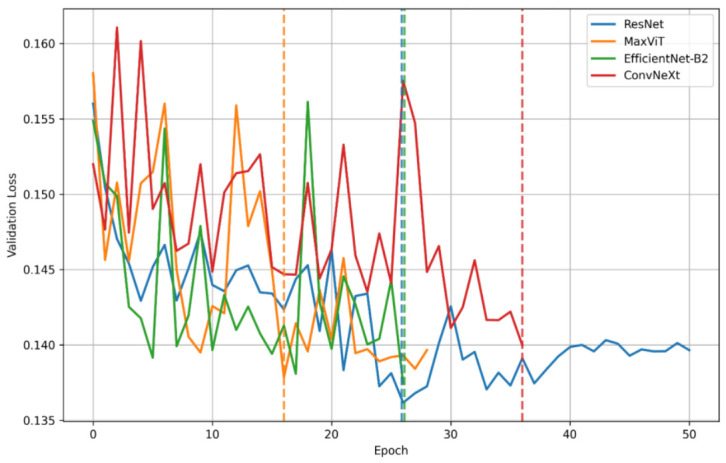
Comparison of validation loss over epochs during the training process for the ResNet, EfficientNet-B2, ConvNeXt, and MaxViT models.

The validation accuracy and validation loss curves from the training process are presented in Figure 12 and Figure 13. No significant divergence between the training and validation curves was observed for any of the models, indicating a low risk of overfitting. In the EfficientNet-B2 and ResNet models, the substantial overlap between training and validation performance further supports the strong generalization capabilities of these architectures. The MaxViT model reached its best validation performance in earlier epochs, demonstrating rapid convergence, whereas the ConvNeXt model exhibited a later and more fluctuating convergence process.

**Figure 14:**
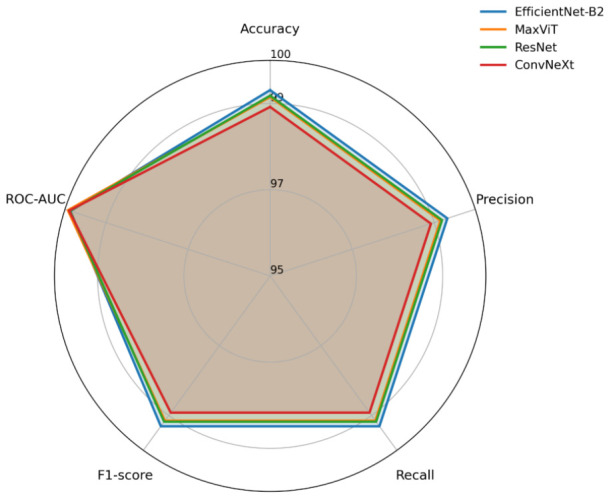
Radar chart showing the performance comparison of the EfficientNet-B2, ResNet, ConvNeXt, and MaxViT models based on accuracy, precision, recall, F1-score, and ROC-AUC metrics.

Examination of the ROC curves and AUC values shows that all models achieved AUC values close to or above 0.99. The radar chart presented in Figure 14 provides a visual summary for comparing the models across different performance metrics. EfficientNet-B2 and MaxViT exhibited ROC curves closest to an ideal classifier, while ResNet and ConvNeXt closely followed with only minimal differences. The high AUC values indicate that the proposed approach can produce reliable results even across different decision thresholds.

Overall, it can be concluded that all the deep learning architectures used in this study can be employed for automatic detection of solar panel cell defects with high accuracy. Among them, the EfficientNet-B2 architecture stands out by demonstrating the most balanced and superior performance in terms of classification accuracy, error distribution, learning stability, and discriminative capability. These findings clearly indicate that the choice of architecture plays a decisive role in system performance and that deep learning based approaches offer a powerful solution for solar panel maintenance and quality control processes.

To ensure the validity of the reported results, dataset splitting was strictly performed at the image level prior to any preprocessing or augmentation. Each image was assigned to only one subset (training, validation, or test), ensuring no overlap between datasets. All preprocessing operations were applied after dataset splitting, and data augmentation was used exclusively for the training set. Therefore, the evaluation results presented in this section are obtained on completely unseen test data, ensuring a fair assessment of model performance.

## 4. Discussion

Recent studies in photovoltaic defect detection using electroluminescence (EL) imaging have increasingly focused on deep learning-based approaches, particularly convolutional neural networks and hybrid transformer architectures. For instance, CNN-based frameworks have demonstrated strong performance in EL defect classification tasks, achieving robust results through improved feature extraction and contrast enhancement techniques [33]. More recent works have introduced transformer and attention-based mechanisms to better capture global contextual information in PV defect regions, further improving detection robustness under complex defect patterns [34]. In addition, hybrid CNN–Transformer architectures and lightweight deep learning models have been proposed to address computational efficiency while maintaining high detection accuracy [35]. Despite these advancements, most existing studies still focus on either limited model families, specific datasets, or single architecture evaluations, and relatively few works provide a unified benchmarking framework across multiple state-of-the-art architectures under identical experimental conditions. This highlights the need for systematic comparative studies that evaluate both classification performance and computational efficiency under controlled settings. This architecture selection enables a comprehensive evaluation of different deep learning paradigms under identical conditions, ensuring a fair comparison across classical, modern, and transformer-based models. In this study, a deep learning-based approach was proposed for binary classification of solar panel cells as defective or normal, and the performance of four modern architectures—EfficientNet-B2, ConvNeXt, MaxViT-T, and ResNet-50—was comparatively evaluated. The results demonstrate clear improvements over previous studies in terms of both overall classification performance and class balance. Beyond a simple comparison of deep learning models, the main novelty of this study lies in establishing a unified binary classification framework for heterogeneous solar cell types and integrating explainability methods for model interpretation.

In the literature, solar cell defect detection has typically relied on classical CNNs or older deep learning architectures, with reported accuracies generally ranging between 95% and 97%, often limited by class imbalance and reduced generalization performance [36,37,38,39]. In this context, the proposed method achieves superior performance, with all evaluated architectures exceeding 98% accuracy and EfficientNet-B2 reaching the highest performance at 99.31%.

Among the evaluated models, EfficientNet-B2 demonstrated the best overall performance in terms of accuracy, precision, recall, and F1-score, while maintaining balanced classification behavior across both classes. MaxViT-T showed fast convergence and stable training behavior with symmetric class-wise performance, whereas ConvNeXt and ResNet-50 also achieved strong but slightly lower results in Table 9.

These results quantitatively demonstrate the trade-off between classification performance and computational cost. EfficientNet-B2 provides the most compact architecture in terms of parameters, FLOPs, model size, and memory usage, making it the most computationally efficient model among those evaluated. Confusion matrix and ROC-AUC analyses confirmed consistently low misclassification rates across all models, indicating strong discriminative capability in detecting fine structural defects such as cracks and fractures [10,30,31].

Heat map visualizations further showed that all models focused primarily on defective regions. EfficientNet-B2 and MaxViT-T produced more localized and precise attention maps, while ConvNeXt and ResNet-50 exhibited more distributed activation patterns. Training and validation curves indicated stable learning behavior with minimal overfitting across all architectures.

Deep learning experiments are inherently subject to experimental variability arising from random weight initialization, mini-batch ordering, data shuffling, data augmentation randomness, and non-deterministic GPU computations. As a result, small variations in performance metrics such as accuracy and F1-score should not be interpreted as definitive evidence of model superiority without proper statistical validation. In this study, all models were trained and evaluated under identical experimental conditions, including the same dataset split, preprocessing pipeline, input resolution, optimizer settings, batch size, and early stopping criteria, ensuring a fair and controlled comparison. Nevertheless, model evaluation is performed using multiple complementary metrics, including accuracy, weighted F1-score, ROC-AUC, confusion matrices, and explainability-based heatmaps, as well as computational complexity measures. This multi-dimensional evaluation reduces the risk of over-interpreting minor metric differences and provides a more robust assessment of model performance.

To determine whether the observed performance differences are statistically meaningful, a significance analysis was performed using EfficientNet-B2 as the reference model. Since all models were evaluated on the same test set, pairwise comparisons were conducted using a two-proportion z-test. The results are summarized below in Table 10.

A limitation of this study is that each model was trained and evaluated using a single experimental run. While this approach ensures a controlled and fair comparison under identical conditions, it does not capture variability arising from random initialization, data shuffling, and non-deterministic GPU operations. Therefore, the reported results represent performance under a fixed experimental configuration rather than averaged performance across multiple runs. This is particularly important for models with close performance values, where small differences in accuracy may not necessarily reflect statistically significant superiority. Future studies should include multiple runs with different random seeds and report mean and standard deviation values to improve statistical robustness.

Although EfficientNet-B2 achieved the highest numerical accuracy (99.31%), the differences compared to other architectures are small (0.13–0.39%). All *p*-values are above 0.05, indicating that these differences are not statistically significant at the 95% confidence level. Therefore, all models exhibit comparable classification capability on EL image data. Therefore, EfficientNet-B2 should be regarded as the highest-performing model under the present experimental configuration rather than being interpreted as universally superior to the other evaluated architectures.

To evaluate computational efficiency and practical applicability, all models were tested under identical conditions using 224 × 224 input resolution on an NVIDIA RTX 3080 Laptop GPU. To evaluate computational efficiency and practical applicability, all models were tested under identical conditions using 224 × 224 input resolution on an NVIDIA RTX 3080 Laptop GPU. In addition to qualitative discussion of efficiency, a quantitative complexity analysis was conducted to provide a more rigorous comparison between architectures. This includes the number of trainable parameters, FLOPs, inference time per image, model size, and peak GPU memory consumption for each model. This extended evaluation allows a more objective assessment of deployment feasibility in real-world photovoltaic inspection systems. The comparison of model complexity is presented below in Table 11.

EfficientNet-B2 demonstrates the lowest parameter count, FLOPs, model size, and memory usage, confirming its superior computational efficiency [16]. Although its inference time is slightly higher than some architectures, it provides the best overall trade-off between accuracy and computational cost.

Another factor contributing to the high performance of all models is the use of ImageNet pre-trained weights. Transfer learning enables models to leverage low-level visual features such as edges, textures, and contrast transitions, which are transferable to electroluminescence imaging and improve convergence and generalization [9,10,11,12,13,16,17]. However, performance gains cannot be attributed solely to transfer learning, as dataset balance, preprocessing, augmentation, and early stopping also contributed significantly. A comparison with randomly initialized models was not performed due to computational constraints and is identified as a limitation for future work.

The dataset design follows a binary classification formulation aligned with practical inspection requirements. The primary objective of this study is not to develop a fine-grained defect subtype classification system, but rather to build a fast, robust, and practical binary decision framework (normal vs. abnormal) that can be directly applied in real-world photovoltaic inspection scenarios. In industrial production lines, quality control systems, and field maintenance applications, the most critical decision is typically whether a solar cell is operationally acceptable or requires further inspection or replacement, rather than distinguishing between specific defect subtypes such as cracks or breaks. From this perspective, both cracked and broken cells represent structurally compromised and performance-degrading conditions and therefore naturally belong to a unified “defective” category.

Furthermore, in electroluminescence (EL) imaging, the visual boundaries between cracked and broken cells are not always clearly separable. Variations in imaging conditions, defect severity, and material properties may lead to overlapping visual characteristics between these two defect types. In such cases, a binary classification strategy improves decision stability by focusing on the most relevant operational distinction: normal versus abnormal.

Nevertheless, extending the proposed framework to a multi-class defect classification setting is straightforward from a methodological perspective. The current architecture can be adapted by modifying the final classification layer to include additional defect categories such as cracked, broken, and other defect types. The preprocessing pipeline, training strategy, and explainability framework can remain unchanged. However, reliable multi-class classification would require a sufficiently large and well-annotated dataset with balanced representation of each defect subtype. Without such conditions, inter class visual ambiguity may lead to reduced classification reliability.

Therefore, the binary classification strategy adopted in this study aligns with practical deployment requirements and ensures robust performance for real-world applications. Multi-class defect classification is considered a natural extension of this work and will be investigated in future studies using more comprehensive and strongly annotated datasets.

Although strict data splitting and data leakage prevention strategies were applied in this study, it should be noted that all experiments were conducted on a single electroluminescence dataset collected under controlled laboratory conditions. Therefore, the reported results reflect strong within-dataset generalization rather than cross-domain or real-world generalization performance. Variations in imaging systems, acquisition settings, and solar cell types may influence model performance in practical applications.

A key limitation of this study is the absence of external validation using independent datasets. Since all experiments were conducted on a single electroluminescence (EL) dataset, the reported results reflect within-dataset generalization rather than cross-domain robustness. Therefore, the performance of the proposed models may vary when evaluated on data acquired from different imaging systems, solar cell types, or photovoltaic production environments, where variations in resolution, contrast, and defect visibility can exist. Grad-CAM visualizations suggest that the models primarily attend to physically meaningful defect regions such as cracks and structural fractures. However, these visualizations should be interpreted as qualitative explanations of model attention and do not, by themselves, exclude the possibility of dataset-specific artifacts or bias. Accordingly, future work will include evaluation on independent datasets collected under different acquisition conditions to better assess real-world generalization capability.

Although the proposed approach achieves high performance, several limitations should be acknowledged. The study is based on a single dataset, which may limit generalization to different imaging conditions or production environments. No external validation dataset was used, and k-fold cross-validation was not performed due to computational cost, although reproducibility was ensured using a fixed random seed. These aspects should be addressed in future studies for stronger validation [29,30,31]. Strict dataset partitioning at the image level, combined with the exclusion of augmented samples from validation and test sets, ensures that the reported performance reflects true generalization rather than memorization of visually similar instances.

A key limitation of this study is the absence of an external validation dataset. Although the proposed framework demonstrates strong performance in terms of accuracy, precision, recall, F1-score, and ROC-AUC, its generalization capability to unseen data from different electroluminescence imaging systems cannot be fully guaranteed. In addition to ROC curve analysis, the numerical ROC-AUC values further confirm that all models achieve near-perfect separability between defective and normal classes, with minimal variation across architectures. The Grad-CAM-based visual explanations suggest that the models focus primarily on physically meaningful defect regions. Nevertheless, Grad-CAM provides qualitative interpretability only and should not be considered definitive evidence for the absence of dataset-specific artifacts or bias. However, since all experiments were conducted on a single EL dataset, variations in imaging devices, acquisition settings, illumination conditions, and solar cell types may affect real-world performance. Therefore, while the results demonstrate strong performance under controlled conditions, future work should include validation on independent datasets collected from different photovoltaic production environments to further assess generalization capability.

## 5. Conclusions

In this study, four deep learning architectures, EfficientNet-B2, ConvNeXt, MaxViT-T, and ResNet-50, were evaluated for the binary classification of solar panel cells as defective or normal using electroluminescence images. All models demonstrated high accuracy, strong generalization capability, and balanced class-wise performance. Among the evaluated models, EfficientNet-B2 achieved the highest numerical accuracy (99.31%) under the present experimental setting and exhibited consistently balanced performance across evaluation metrics. However, given that each architecture was trained and evaluated using a single experimental run, these differences should not be interpreted as definitive evidence of architectural superiority. However, statistical significance analysis indicated that the performance differences between EfficientNet-B2 and the other evaluated architectures were not significant at the 95% confidence level. MaxViT-T exhibited fast convergence, stable training behavior, and highly balanced classification performance across classes. ResNet-50 provided a strong and stable baseline, while ConvNeXt achieved competitive accuracy with minor fluctuations in validation performance. Quantitative results, including confusion matrices, ROC-AUC analysis, and heat map visualizations, confirmed consistently low misclassification rates and reliable detection of fine-grained structural defect patterns. In particular, EfficientNet-B2 and MaxViT-T showed more focused attention on defective regions, whereas ConvNeXt and ResNet-50 exhibited relatively broader attention distributions. Training and validation curves further indicated minimal overfitting and strong generalization performance across all models. Beyond model comparison, this study introduces a unified binary defect detection framework and demonstrates its effectiveness across multiple state-of-the-art architectures. A limitation of this work is the absence of external dataset validation, which should be addressed in future studies to further evaluate generalization under different acquisition conditions. In addition, cracked cells represent the most challenging defect category due to their subtle structural variations and low contrast in electroluminescence images. In addition, another important limitation of this study is that each model was trained and evaluated using a single experimental run. Due to the stochastic nature of deep learning training—such as random weight initialization, mini-batch ordering, data shuffling, and non-deterministic GPU operations—the results may vary slightly across different runs. Therefore, although identical training settings were applied to all models to ensure fairness, the reported results should be interpreted as performance under a single controlled run rather than statistically averaged performance. This may particularly affect the interpretation of small performance differences between models. Consequently, the observed differences in accuracy, F1-score, and ROC-AUC should not be considered definitive evidence of architectural superiority without additional statistical validation.

## Figures and Tables

**Table 1:** Composition of the electroluminescence (EL) image dataset used in this study.

Category	Sub-Category	Number of Images
Total Samples	All EL Images	20,400
Cell Condition	Normal	10,200
	Defective	10,200
Defect Type	Cracked	7225
	Broken	2975
Cell Type	Monocrystalline	11,852
	Polycrystalline	8548

**Table 2:** Software environment and training configuration.

Parameter	Value
Programming Language	Python 3.12.4
Deep Learning Framework	PyTorch 2.5.1
CUDA Version	CUDA 12.1
GPU	NVIDIA GeForce RTX 3080 Laptop GPU
Input Image Size	224 × 224
Batch Size	32
Maximum Epochs	250
Optimizer	AdamW
Learning Rate	3 × 10^−4^
Weight Decay	1 × 10^−4^
Loss Function	Cross-Entropy Loss
Label Smoothing	0.05
Learning Rate Scheduler	CosineAnnealingLR
Pretrained Weights	ImageNet
Early Stopping	Validation loss-based
Patience	12 epochs
Minimum Epochs	30
Data Augmentation	Training set only
Validation/Test Augmentation	None
Evaluation Metrics	Accuracy, Precision, Recall, F1-score, ROC-AUC, Confusion Matrix

**Table 3:** Hardware configuration and model-specific training time.

Model	GPU	Batch Size	Input Size	Best Epoch	Total Training Time
EfficientNet-B2	NVIDIA GeForce RTX 3080 Laptop GPU	32	224 × 224	27	45 min
ResNet-50	NVIDIA GeForce RTX 3080 Laptop GPU	32	224 × 224	21	51 min
ConvNeXt-Tiny	NVIDIA GeForce RTX 3080 Laptop GPU	32	224 × 224	37	59 min
MaxViT-T	NVIDIA GeForce RTX 3080 Laptop GPU	32	224 × 224	17	1 h 09 min

**Table 4:** Overall classification performance of deep learning models on the test dataset.

Model	Accuracy (%)	Precision	Recall	F1-Score	ROC-AUC
EfficientNet-B2	99.31	0.9932	0.9932	0.9931	0.9987
ConvNeXt-Tiny	98.92	0.9893	0.9893	0.9892	0.9990
MaxViT-T	99.15	0.9915	0.9915	0.9915	0.9993
ResNet-50	99.18	0.9919	0.9918	0.9918	0.9986

**Table 5:** Precision, recall, and F1-score of the EfficientNet-B2 model on the test dataset.

Class	Precision	Recall	F1-Score	Support
Defective	0.9974	0.9889	0.9931	1530
Normal	0.9890	0.9974	0.9932	1530
Accuracy			0.9931	3060
Macro Avg	0.9932	0.9931	0.9931	3060
Weighted Avg	0.9932	0.9931	0.9931	3060

**Table 6:** Precision, recall, and F1-score of the ConvNeXt model on the test dataset.

Class	Precision	Recall	F1-Score	Support
Defective	0.9921	0.9863	0.9892	1530
Normal	0.9864	0.9922	0.9892	1530
Accuracy			0.9892	3060
Macro Avg	0.9892	0.9892	0.9892	3060
Weighted Avg	0.9892	0.9892	0.9892	3060

**Table 7:** Precision, recall, and F1-score of the ResNet-50 model on the test dataset.

Class	Precision	Recall	F1-Score	Support
Defective	0.9967	0.9869	0.9918	1530
Normal	0.9871	0.9967	0.9919	1530
Accuracy			0.9918	3060
Macro Avg	0.9919	0.9918	0.9918	3060
Weighted Avg	0.9919	0.9918	0.9918	3060

**Table 8:** Precision, recall, and F1-score of the MaxViT model on the test dataset.

Class	Precision	Recall	F1-Score	Support
Defective	0.9915	0.9915	0.9915	1530
Normal	0.9915	0.9915	0.9915	1530
Accuracy			0.9915	3060
Macro Avg	0.9915	0.9915	0.9915	3060
Weighted Avg	0.9915	0.9915	0.9915	3060

**Table 9:** Comparison of classification performance of deep learning models on the test dataset.

Model	Accuracy (%)	Precision	Recall	F1-Score
ResNet-50	99.18	0.9919	0.9918	0.9918
EfficientNet-B2	99.31	0.9932	0.9932	0.9931
ConvNeXt-Tiny	98.92	0.9893	0.9893	0.9892
MaxViT-T	99.15	0.9915	0.9915	0.9915

**Table 10:** Statistical significance analysis of accuracy differences using EfficientNet-B2 as reference model.

Comparison	Accuracy Difference	Test	*p*-Value	Interpretation
EfficientNet-B2 vs. ConvNeXt-Tiny	0.0039	Two-proportion z-test	0.101	Not significant
EfficientNet-B2 vs. MaxViT-T	0.0016	Two-proportion z-test	0.464	Not significant
EfficientNet-B2 vs. ResNet-50	0.0013	Two-proportion z-test	0.256	Not significant

**Table 11:** Computational complexity and efficiency comparison of architectures.

Model	Parameters (M)	FLOPs (G)	Inference Time (ms)	Model Size (MB)	GPU Memory (MB)
EfficientNet-B2	7.704	0.681	24.010	29.82	48.43
ConvNeXt-Tiny	27.822	4.470	8.904	106.20	128.26
MaxViT-T	30.409	5.608	30.344	116.83	159.35
ResNet-50	23.512	4.109	8.411	90.00	110.75

## Data Availability

The data available on request due to restriction (privacy, legal or ethical reason).
